# Ticking the boxes: a survey of workplace-based assessments

**DOI:** 10.1192/pb.bp.114.049098

**Published:** 2016-04

**Authors:** Thomas Gilberthorpe, Maame Duku Sarfo, Geoff Lawrence-Smith

**Affiliations:** 1 South London and Maudsley NHS Foundation Trust; 2 Surrey and Borders Partnership NHS Foundation Trust; 3 Oxleas NHS Foundation Trust

## Abstract

**Aims and method** To survey the quality of workplace-based assessments (WPBAs) through retrospective analysis of completed WPBA forms against training targets derived from the Royal College of Psychiatrists' Portfolio Online.

**Results** Almost a third of assessments analysed showed no divergence in assessment scores across the varied assessment domains and there was poor correlation between domain scores and the nature of comments provided by assessors. Of the assessments that suggested action points only half were considered to be sufficiently ‘specific’ and ‘achievable’ to be useful for trainees' learning.

**Clinical implications** WPBA is not currently being utilised to its full potential as a formative assessment tool and more widespread audit is needed to establish whether this is a local or a national issue.

Workplace-based assessment (WPBA) was introduced as part of Modernising Medical Careers (MMC) and became the principal formative assessment method of the competency-based curriculum implemented by the Royal College of Psychiatrists in 2007.^1,2^ As a result, progression through training is now dependent on the achievement of defined competencies outlined in the College curriculum and demonstrated using an assessment form covering areas such as Case-based Discussion (CbD), Assessment of Clinical Expertise (ACE) and mini-Assessed Clinical Encounter (mini-ACE). Completion of a specified number of WPBAs is a mandatory requirement of the Annual Review of Competence Progression (ARCP) for trainees before they can progress to the next year of training.^3^

Since its introduction, WPBA has come under considerable scrutiny and questions have been raised about its appropriateness and effectiveness as a performance assessment tool.^4^ A number of studies published in the *BJPsych Bulletin* since 2009 have revealed that both trainees and assessors perceive WPBA in a less than favourable light and remain unconvinced of its effectiveness as a tool for measuring performance and improving the quality of psychiatric training.^5-7^ However, it should be borne in mind that such studies are qualitative and rely heavily on self-reports, which may be subject to negative recall bias.

Despite these concerns, it appears that no studies have been conducted that directly examine the content of completed assessments to inform whether or not they are being utilised in the manner they were intended to be in accordance with the College guidance. Our survey aimed to address this discrepancy by examining current practice in the completion of WPBA by assessors within Oxleas NHS Foundation Trust and comparing this against the guidance set out by the College.

Although there are no explicit standards published for assessors with regard to the completion of WPBAs, suggestions are made in the online trainees' guide on how to conduct an assessment.^8^ In light of this we refer to ‘training targets’ or ‘educational targets’ rather than explicit standards, a position currently adopted by the College's Prescribing Observatory for Mental Health-UK (POMH-UK) who use treatment targets when no such standards exist or when standards are not easily measurable.^9^

## Method

Our first task was to arrive at appropriate training targets against which we could then measure our data-set. With reference to information published on the College's Portfolio Online we derived a number of WPBA training targets:

- evidence of divergence in scores across the assessment domains as opposed to identical scores awarded across the whole assessment
- evidence of assessors using the unable to comment (U/C) score when appropriate
- evidence of completion of all assessment comment boxes
- evidence of correlation between written feedback and domain scores
- provision of action points that suggest to the trainee realistic ways to address learning points arising from the WPBA, in line with the SMART (Specific, Measurable, Achievable, Relevant and Timely) method of goal development.^10^

We subsequently identified all core and higher psychiatry trainees on the Oxleas rotation between February and August 2013 and approached them for written consent to use their WPBA forms in the survey. We provided each trainee with an information leaflet explaining the nature and purpose of the audit. Of a total number of 17 trainees, 13 provided signed written consent and 4 provided written consent in the form of an email communication. Once consent was given, we collected our raw data by meeting with trainees to print out completed WPBAs from their online portfolios.

Data were first analysed separately by two of the authors who then conferred together to ensure internal consistency and reliability; the analysis was done using Microsoft Excel. The nature of the comments as pertaining or not to the assessment scores and whether the action points were specific and achievable were also investigated separately and any discrepancies of opinion were discussed to arrive at a consensus decision. In our subjective opinion, an example of a ‘specific’ and ‘achievable’ action point would be for a trainee to revise the aetiology of schizophrenia by reading a relevant article in *BJPsych Advances* for discussion with the clinical supervisor on a (preferably) specified date.

## Results

We collected a total of 124 WPBAs for analysis (30 ACEs, 47 mini-ACEs and 47 CbDs). Of those, 47 assessments were at CT1 level, 24 were at CT2 level, 22 at CT3 level and 31 at ST4-6 level. The vast majority of assessors were senior medical staff with 95 assessments completed by consultant psychiatrists and 18 by specialist registrars and staff grades. The remainder was completed by nurses (*n* = 6) and clinical psychologists (*n* = 5).

### Assessment of ‘domain scores’

Our sample of 124 WPBAs yielded a total number of 1086 domain scores for analysis. For mini-ACEs and ACEs, 8 domains or areas of competence are assessed using a score from 1 to 6. For CbDs, the number of domains assessed is 10. The U/C ‘score’ is used if the assessor feels unable to comment on a particular domain. The U/C score might conceivably be given in the domain of ‘clinical record-keeping’ following a purely face-to-face clinical encounter where no documentation was available for review by the assessor. A score between 1 and 3 indicates that a trainee's performance in a particular competence is below what is expected for their current training level; a score of 4 meets the expected standards; and scores of either 5 or 6 indicate performance at a standard higher than expected.

**Fig. 1:**
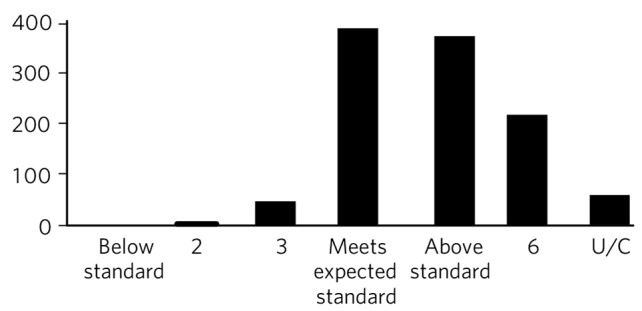
Frequency of domain scores on Assessment of Clinical Expertise/mini-Assessed Clinical Encounter/Case-based Discussion, total sample (*n* = 124 assessments). U/C, unable to comment.

Overall, 90% of domain scores were 4 or above: 36% were 4s, 34% were 5s and 20% were 6s. Accordingly, there were very few domain scores below 4 (3.2%) and none of the trainees were given a score of 1 in any domain across the whole sample. Around 5% of domains were not scored and were marked as unable to comment (U/C) (Fig. 1).

**Fig. 2:**
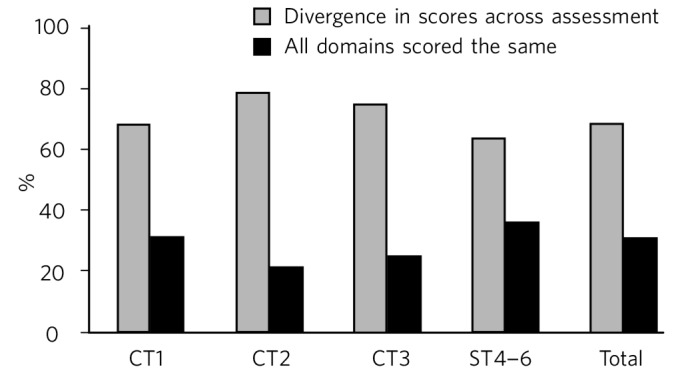
Comparison of divergence in scores by at least one domain against identical scores given across the whole assessment.

From our data-set, in 39 out of 124 assessments (31%), all domains were scored the same (Fig. 2), creating a ‘straight line’ of scores across the assessment, a phenomenon we have denoted the ‘straight-line effect’. The potential significance of such a straight-line effect might be to differentiate between assessors who actively engage in the assessment process as opposed to treating it as a tick-box exercise. Of the 39 assessments with no variation in scores, 29 were completed by consultant psychiatrists. Certain consultant assessors were more prone to lack divergence in their scoring, with 62% of non-divergent assessments being completed by 3 out of 23 consultant assessors. Only 3 out of 18 assessments completed by specialist registrars and staff grades showed a straight-line effect. Half of assessments completed by nursing staff and 4 out of 5 assessments by psychologists showed no variation in domain scores.

In only 44% of assessments any further details of the clinical case were provided for future reference and reflective practice. Only 46% of assessments demonstrated an obvious correlation between feedback comments and domain scores. Of 124 assessments, 17 (14%) contained scores below 4, and of these 10 (59%) provided comments that specifically addressed these low scores. Of the 95 (77%) assessments with a completed comment on ‘agreed action’, 48 (51%) were considered to stipulate specific and achievable goals for the trainee's further professional development. In only 8% of assessments was no written feedback provided at all.

## Discussion

The results of our audit provide important information about the ways in which WPBA assessments are being completed in Oxleas NHS Foundation Trust and we suspect that the results may well be broadly applicable to other trusts.

First, it is reassuring to note that consultants, particularly clinical supervisors, carry out the majority of assessments as this is a requirement of the ARCP. However, further efforts need to be made in training and utilising other professionals in conducting WPBAs. Second, although at first glance our results may suggest that WPBA is being carried out to an acceptable standard, there are indications that WPBA is not being utilised as effectively as it could be as a formative assessment tool. A concern highlighted in previous literature about WPBA tools was that it was easy for assessors to simply tick all domain boxes with the same score creating the ‘straight-line effect’ we have referred to earlier. The absence of any divergence in scores across entire assessments may reflect such a lack of engagement in the formative assessment process on the part of the assessors.

From our data, almost a third of assessments showed identical domain scores throughout and only 5% of all domains scores were marked as U/C. Over the course of this survey, we have come to consider the U/C box as akin to the ‘trick questions’ on some rating scales, used to increase validity. We are sceptical that any one WPBA is sufficiently broad in scope or duration for all domains to be adequately assessed (e.g. the assessment of clinical record-keeping in a purely face-to-face clinical encounter). We assert that the inability to assess certain domains at one WPBA has utility in itself by serving to explicitly highlight gaps in trainee experience that need to be addressed in subsequent assessments.

Another important observation is the fact that there is a significant skewed distribution in scores across the dataset, with only 3.2% of domain scores being a 3 or lower. One possible explanation is that the trainees in our sample were exceptionally competent across the board and this finding truly reflects their high standards. However, another explanation is that many assessors are reluctant to mark trainees down. This may reflect both a lack of assessors' appreciation of the formative nature of WPBAs and familiarity with the scoring criteria. Assessors may also lack the confidence to give a lower score because of the perceived negative impact such scores may have on the trainee's confidence.

Given that one of the main stated aims of WPBA is to provide constructive feedback on performance, the provision of written comments and feedback is essential. In only 47% of assessments were further details of the clinical case provided. Although it is important to retain patient confidentiality, some contextual information about the case would serve the trainee well for future reflection as they progress through training. Other medical Royal Colleges such as the Royal College of General Practitioners and the Royal College of Ophthalmologists use assessment tools for this explicit purpose.^11,12^ In the case of the Royal College of Ophthalmologists, the trainee completes the background details of the WPBA, including details of the case and the issue that was discussed or assessed. The assessor then has to score the trainee and edit, reject or approve the assessment. This allows relevant details pertaining to the case in question to be entered in the WPBA, facilitating subsequent reflective learning. It also reduces the time required from the assessor, which in previous literature has been cited as one of the main obstacles to WPBA.

It is noteworthy that only 46% of the assessments conducted showed clear correlation between comments noted and domain scores given, something that would be helpful for trainees to both acknowledge areas for improvement (e.g. clinical record-keeping) and to be mindful of what they are doing well (e.g. risk assessment).

In just over half (51%) of assessments which provided agreed action points did we consider those points to be sufficiently specific and achievable to be helpful for the trainee's onward learning. At a more senior level, the annual appraisal process encourages clearly defined objectives for personal and professional development and we feel that the WPBA should be a reflection of this, providing strategies for reflective learning that can be reviewed at a later date.

The findings of our audit suggest the need for further training and guidance on the use of WPBA in order for it to be used most effectively for what it was originally intended, as primarily a formative rather than summative assessment. To achieve this we suggest a number of local interventions:

- creating a series of video vignettes to address areas of good and poor performance on the part of the trainers/assessors which can complement the training already provided by the College
- setting up of focus groups of trainers and trainees together to explicitly identify where improvements to the practice of formative assessment can be made and to manage the expectations of trainees and trainers alike in the area of WPBA
- paying greater attention to WPBA in the educational supervisors' appraisal every 3 years using data from surveys such as this one
- broadening the provision of training in WPBA to colleagues from disciplines outside medicine (e.g. psychology and nursing) to ensure parity in standards of assessment for our trainees.

At a broader level, we would question the utility of having such specific scoring criteria for WPBAs and suggest that a simpler ‘competent, excellent, need to improve’ system be employed in the assessment of psychiatric trainees in the workplace.

We appreciate the reality of clinicians' time constraints and that this has been a criticism of WPBA in the past. However, we feel that with enhanced training in the specific use of WPBA for formative purposes the process can be made more time-efficient and educationally effective. To aid this, we would recommend that more specific assessment standards for trainers be published in the College's Portfolio Online. Furthermore, the majority of assessors are consultants who as clinical supervisors should devote an hour every week for supervision, which is enough time to carry out a thorough assessment with feedback. The key is to plan and schedule these assessments early in the placement to ensure they are spread out and do not pile up at the end.

### Limitations of the study

This is a simple baseline survey, which would need to be repeated once our proposed interventions have been implemented in order for it to become a completed audit cycle. Furthermore, there are no explicit standards against which to compare local practice so we have had to arrive at these ourselves using the guidance published on the College's Portfolio Online. It should be noted that the judgement of whether action points were specific and achievable was based on our subjective opinion and not on a validated measuring tool.

We are aware that our sample size is small, involving only 17 trainees, 37 assessors and 124 assessments, and concede that the significance of our conclusions remains uncertain.

### Further research

We propose that this survey is repeated in other mental health trusts to determine whether our findings reflect national trends or a local cohort effect specific to Oxleas. This would be practically straightforward and manageable to undertake. Such a survey could be carried out robustly under the auspices of the Royal College of Psychiatrists, given its credible and prime position to coordinate this on a large scale at a national level. Other useful follow-up work in this area might be of a qualitative nature looking more closely at the content of the written feedback provided in WPBAs and ascertaining whether or not this was deemed or felt to be useful or not by trainees themselves.
